# A Mucosal Change like Hypertrophic Gastritis Following Zolbetuximab-Based Therapy in a Conversion Surgery Case of Advanced Gastric Cancer

**DOI:** 10.3390/reports8040235

**Published:** 2025-11-13

**Authors:** Soshi Oyama, Shuhei Suzuki, Takanobu Kabasawa, Takumi Kanauchi, Shotaro Akiba

**Affiliations:** 1 Department of Internal Medicine, Yamagata Prefectural Shinjo Hospital, Shinjo 996-8585, Japan; 2 Department of Pathology, Yamagata University School of Medicine, Yamagata 990-9585, Japan

**Keywords:** zolbetuximab, gastric cancer, claudin 18.2, hypertrophic gastritis, conversion surgery

## Abstract

**Background and Clinical Significance**: Zolbetuximab, a claudin 18.2-targeted monoclonal antibody, has demonstrated efficacy in advanced gastric cancer. Hypoalbuminemia has emerged as a notable adverse effect, but its underlying mechanism remains unclear. **Case Presentation**: A 53-year-old male with unresectable advanced gastric cancer received zolbetuximab-based combination therapy, achieving tumor regression enabling conversion surgery. During six cycles of treatment, serum albumin levels decreased from 4.3 g/dL to 3.5-3.6 g/dL (grade 1 hypoalbuminemia). A histopathological examination of the surgical specimen revealed hypertrophic gastritis characterized by marked foveolar hyperplasia, increased mucus secretion, and pyloric gland metaplasia on the lesser curvature. These findings suggest that zolbetuximab-induced mucosal alterations may contribute to hypoalbuminemia through enhanced protein loss. **Conclusions**: This is the first pathological documentation of hypertrophic gastritis associated with zolbetuximab therapy. Clinicians should monitor albumin levels during treatment and consider nutritional support when indicated. These findings provide important insights for optimizing patient management and ensuring safe conversion surgery planning.

## 1. Introduction and Clinical Significance

Gastric cancer remains one of the most prevalent malignancies in Japan, with approximately 20% of patients diagnosed at Stage IV [1]. The 5-year survival rate for these patients is extremely low at only 7% [2], making effective chemotherapy crucial for medical oncologists. Treatment options for unresectable advanced gastric cancer have expanded in recent years. Beyond the standard combination of fluoropyrimidines and platinum agents, improved outcomes have been achieved by incorporating targeted therapies based on biomarker testing, including trastuzumab [3], nivolumab [4], and pembrolizumab [5].

Claudins are essential proteins that form tight junctions between cells. The subtype Claudin 18.2 (CLDN18.2) plays a significant role in gastric tissue [6,7], but it does not affect treatment outcomes with either chemotherapy or immune checkpoint inhibitors [8]. CLDN18.2-targeted CAR-T cell therapies are being developed for the treatment of gastric and gastroesophageal junction cancers [9]. During carcinogenesis, CLDN18.2 becomes exposed on the cell surface [10,11], making it an attractive therapeutic target for gastric cancer. Chemotherapy incorporating zolbetuximab, a specific antibody against CLDN18.2, received insurance approval in 2024 [12,13]. While nausea and vomiting are the well-known adverse effects of zolbetuximab, hypoalbuminemia has recently gained attention [12,13]. Inflammatory changes in the stomach have been postulated as a potential cause, but detailed examinations in humans are lacking. We report a case where after achieving response with zolbetuximab and performing conversion surgery, we found histopathological evidence of hypertrophic gastritis, not merely inflammatory changes. As zolbetuximab is a recently approved drug, this observation provides valuable insight for managing hypoalbuminemia and ensuring safe conversion surgery.

## 2. Case Presentation

A 53-year-old man with a history of cecal surgery and positive hepatitis B surface antigen (HBs antigen) was referred to our hospital after an abnormality was detected during an upper gastrointestinal series screening in his residential area. At presentation, his performance status was excellent (ECOG PS 0), with no weight loss or abdominal symptoms. His body mass index was 23.5 kg/m^2^, and nutritional status was well-maintained with normal serum albumin (4.3 g/dL) and total protein levels. He had no history of chronic gastritis, peptic ulcer disease, or previous gastroscopy. The patient had been asymptomatic until the screening examination revealed the gastric abnormality. He consumed approximately 700 mL of beer daily and was a current smoker with a 33-year history of smoking 10 cigarettes per day. He worked in a factory and had no significant family history.

**Figure 1:**
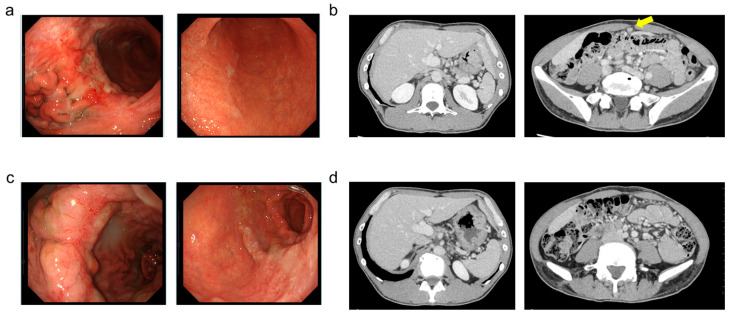
Radiological and endoscopic images. (**a**) Endoscopic images (before chemotherapy). (**b**) Computed tomography images (before chemotherapy)—arrow: peritoneal dissemination. (**c**) Endoscopic images (after chemotherapy). (**d**) Computed tomography images (after chemotherapy). (Improvement in gastric wall thickness from approximately 10–18 mm before treatment to 6–14 mm after chemotherapy).

**Figure 2:**
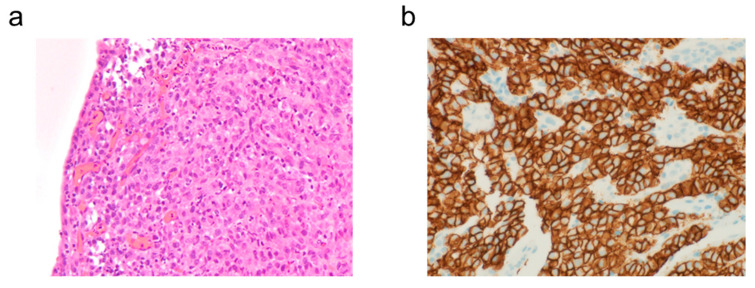
Pathological findings before chemotherapy. (**a**) Endoscopic biopsy specimen; hematoxylin and eosin staining, original ×100. (**b**) Endoscopic biopsy specimen; immunohistochemical staining for CLDN18.2, ×100.

Laboratory data is shown in Table 1. Upper gastrointestinal endoscopy revealed a type 3 tumor in the middle portion of the greater curvature of the gastric body (Figure 1a). Biopsy confirmed poorly differentiated tubular adenocarcinoma (Figure 2a). CT scan suggested peritoneal dissemination (Figure 1b), so after a multidisciplinary conference between internal medicine and surgery departments, systemic chemotherapy was recommended instead of surgery.

Given the patient’s HBs antigen positivity, tenofovir was initiated after confirming negative DNA results. While awaiting the biomarker test results, which were delayed due to regional factors, S-1 (tegafur/gimeracil/oteracil) plus oxaliplatin therapy was started at the patient’s request for prompt treatment initiation.

Biomarker testing revealed human epidermal growth factor receptor type 2 (HER2) negativity and PD-L1 negativity with a Combined Positive Score (CPS) of 1–10% using PD-L1 IHC 22C3 pharmDx (Dako, Copenhagen, Denmark) and 1–5% using PD-L1 IHC 28-8 pharmDx (Dako). Microsatellite instability high (MSI-High) was not detected. CLDN18.2 was positive (Figure 2b). Based on these results, treatment was switched to capecitabine + oxaliplatin + zolbetuximab from the subsequent cycle.

**Figure 3:**
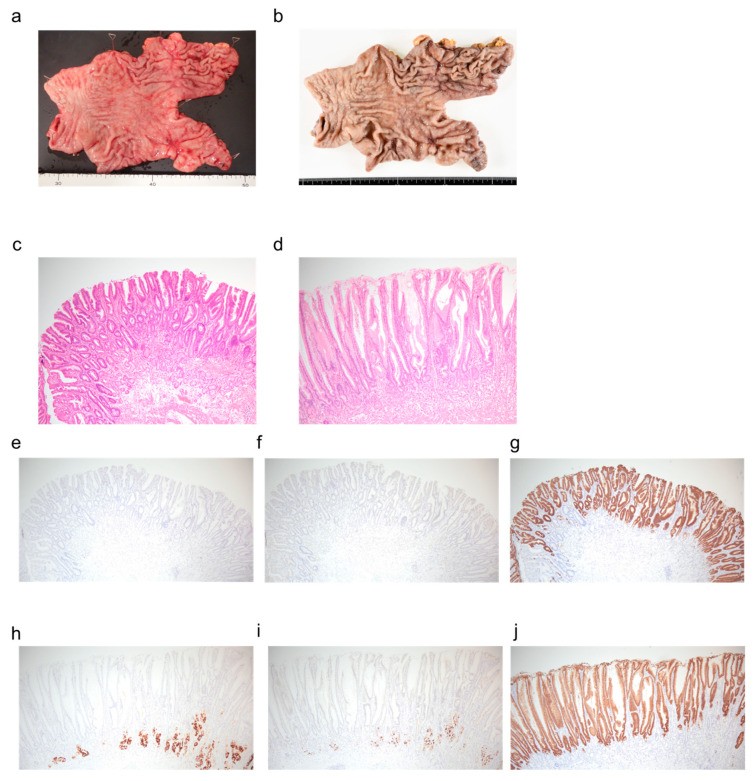
Pathological findings after chemotherapy of surgical specimen. (**a**) Gross photograph of gastric specimen, mucosal aspect (before fixation). (**b**) Gross photograph of gastric specimen, serosal aspect (after fixation). (**c**) Lesser curvature, hematoxylin and eosin staining, ×40. (**d**) Greater curvature, hematoxylin and eosin staining, ×40. (**e**) Lesser curvature, immunohistochemical staining for pepsinogen I, ×40. (**f**) Lesser curvature, immunohistochemical staining for H-K-ATPase, ×40. (**g**) Lesser curvature, immunohistochemical staining for MUC5AC, ×40. (**h**) Greater curvature, immunohistochemical staining for pepsinogen I, ×40. (**i**) Greater curvature, immunohistochemical staining for H-K-ATPase, ×40. (**j**) Greater curvature, immunohistochemical staining for MUC5AC, ×40.

The main adverse events included nausea and grade 1 hypoalbuminemia according to the Common Terminology Criteria for Adverse Events (CTCAE) version 5.0. Follow-up endoscopy and CT (Figure 1c,d) showed significant tumor reduction, and peritoneal dissemination had diminished to the point of being undetectable on imaging. After multidisciplinary discussion, conversion surgery was proposed and accepted by the patient. After completing six cycles of chemotherapy and confirming negative peritoneal cytology, chemotherapy was suspended during a four-week preoperative interval to allow for surgical preparation, after which distal gastrectomy with D2 lymph node dissection and Roux-en-Y reconstruction was performed (Figure 3a,b).

Histopathological evaluation confirmed a therapeutic effect of grade 2b. The macroscopic classification was 0-IIa, measuring 14 mm, with a depth of invasion of ypT1a(M). The histological type was predominantly conventional moderately differentiated tubular adenocarcinoma (tub2) with some well-differentiated tubular adenocarcinoma (tub1). No lymphovascular invasion (Ly0, V0) was observed, and all dissected lymph nodes were negative.

Histological examination (Figure 3c–j) revealed foveolar hyperplasia on both the lesser and greater curvatures, suggesting increased gastric mucus secretion. Additionally, pyloric gland metaplasia was observed on the lesser curvature, while on the greater curvature, the atrophy of the fundic glands was present but mild. Inflammatory cell infiltration was also observed to be mild in degree. These findings most closely matched the pathological entity of hypertrophic gastritis.

The postoperative course was uneventful, with no anastomotic complications. Chemotherapy was resumed on postoperative day 34. At the four-month postoperative follow-up, oxaliplatin was omitted due to grade 1 peripheral neuropathy, but no evidence of recurrence was observed.

## 3. Discussion

Zolbetuximab has recently been added as a treatment option for unresectable advanced gastric cancer, showing significant improvements in progression-free survival and overall survival in CLDN18.2-positive cases [12,13]. It is increasingly being used in clinical practice. CLDN18.2 positivity was higher in EBV-associated and PD-L1-positivity but lower in HER2 positivity [14].

The primary adverse effects of zolbetuximab are severe nausea and vomiting, commonly managed with dexamethasone, 5-HT3 receptor antagonists, neurokinin 1 receptor antagonists, and olanzapine and histamine H1 receptor antagonists [15].

**Figure 4:**
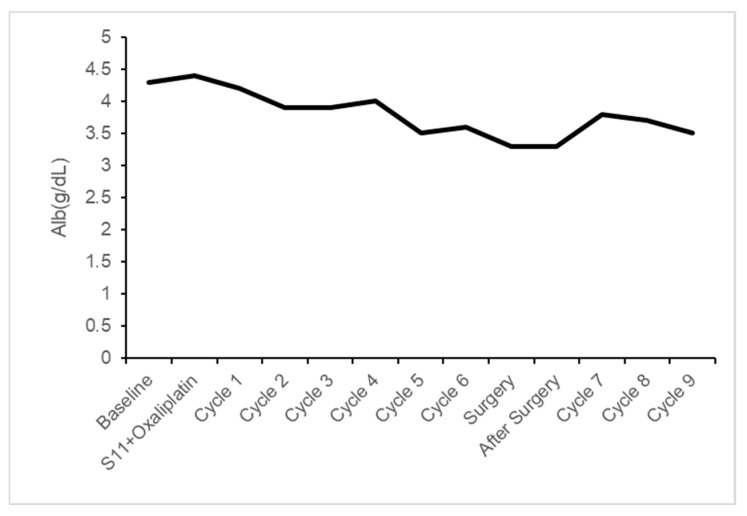
Changes in serum albumin levels.

While focus has been placed on nausea, vomiting, and infusion reactions, hypoalbuminemia [16] has garnered attention as more patients receive zolbetuximab post-marketing. An integrated analysis of the SPOTLIGHT and GLOW trials [17] showed all-grade hypoalbuminemia in 19.3% of the zolbetuximab group versus 10.1% in the placebo group, with grade 3 or higher events occurring in 3.8% versus 1.1%, respectively. Our patient also experienced a decrease in serum albumin levels, albeit grade 1 (Table 2 and Figure 4).

Although inflammatory changes in the stomach are suspected to contribute to hypoalbuminemia, detailed pathological examinations using actual specimens have not been previously reported. Moreover, endoscopic observations alone may be insufficient for evaluating gastric inflammation following zolbetuximab administration. In our case, preoperative endoscopy showed the closure of the ulcer in the tumor area, but pathologically, changes were observed not only in the superficial layer but also in deeper layers. This suggests that comprehensive pathological evaluation using surgical specimens encompassing all layers might be important.

In our case, while the pepsinogen I- and H-K-ATPase-positive fundic glands on the greater curvature were slightly atrophic, the MUC5AC-positive gastric epithelium was well-developed on both the lesser and greater curvatures. These immunohistochemical findings are clinically significant for several reasons. First, the preservation of the MUC5AC-positive foveolar epithelium, despite mild fundic gland atrophy, indicates that the surface mucus-secreting function remains intact, which is essential for maintaining the gastric mucosal barrier. Second, the discrepancy between the slight atrophy of acid-secreting fundic glands (demonstrated by pepsinogen I and H-K-ATPase staining) and the well-preserved surface epithelium suggests a selective or early-stage pathological process rather than advanced pan-mucosal atrophy. This pattern helps distinguish our case from severe atrophic gastritis or intestinal metaplasia, where both glandular and surface epithelial components are typically compromised. Understanding these immunohistochemical markers allows clinicians to better assess the extent and nature of gastric mucosal changes, which has implications for prognosis and management strategies. The glandular tissue on the lesser curvature was pepsinogen I-negative, indicating pyloric gland metaplasia. These findings suggest hypertrophic gastritis, which, to our knowledge, has not been previously reported in association with zolbetuximab, making this observation particularly valuable. No evidence of giant folds suggestive of Ménétrier’s disease or signs of infectious etiology such as cytomegalovirus were observed. Additionally, differential diagnoses that could account for mucosal hypertrophy, such as Zollinger–Ellison syndrome, or those characterized by prominent foveolar hyperplasia, such as hyperplastic polyps, were considered but not supported by the histopathological findings.

The mechanism behind this remains speculative due to a lack of previous reports. Since CLDN18.2 is a tight junction protein expressed in gastric epithelial cells, zolbetuximab might inhibit tight junction structure and function, causing mucosal damage. The resulting repair response could potentially lead to epithelial cell hyperplasia. We speculate that the disruption of tight junctions may alter the gastric microenvironment, affecting local metabolism and immune homeostasis, which could contribute to the hypertrophic changes observed. Additionally, the increased mucus secretion and foveolar hyperplasia might represent compensatory protective mechanisms in response to compromised mucosal barrier function. Hypertrophic gastritis, apart from conditions like Ménétrier’s disease (giant hypertrophic gastritis), is rare, with few other causes such as plasmacytoma [18]. The pathogenesis of hypertrophic gastritis itself requires further research.

Notably, in our first case, due to the therapeutic effect on the tumor, we did not observe findings suggestive of hypertrophic gastritis when comparing pre- and post-treatment CT and upper endoscopy images regarding gastric wall thickness, surrounding invasion, or macroscopic inflammatory changes in the gastric mucosa. However, as the differentiation between type 4 gastric cancer and hypertrophic gastritis is clinically important and challenging [19], attention to pseudoprogression is necessary from a clinical oncology perspective.

A major limitation of this single case report is the absence of pre-treatment full-thickness pathological specimens for comparison. While pre-treatment endoscopic biopsies confirmed adenocarcinoma, these superficial samples cannot be directly compared to the post-treatment surgical specimens. Therefore, we cannot definitively prove that the observed hypertrophic changes were treatment-induced rather than pre-existing or related to tumor regression. Additionally, the findings were observed approximately four weeks after the final zolbetuximab administration due to necessary perioperative discontinuation, which may have modified the clinical presentation. Concomitant medications such as capecitabine, oxaliplatin, and tenofovir could also have influenced the findings. However, to our knowledge, these drugs have not been associated with hypertrophic gastritis, making zolbetuximab the most likely causative agent. As a single case report, our findings represent an association rather than definitive causation, and the possibility of coincidental findings cannot be entirely excluded. The relationship between these pathological changes and hypoalbuminemia, as well as potential preventive approaches, remains to be clarified through the accumulation of additional cases and further investigation.

## 4. Conclusions

In conclusion, we report a case of hypertrophic gastritis observed in a surgical specimen from a patient who achieved good therapeutic response with zolbetuximab. The further accumulation of similar findings and related knowledge is desirable for the effective clinical application of zolbetuximab in advanced gastric cancer treatment.

## Figures and Tables

**Table 1:** Laboratory findings at time of first visit.

Biochemistry			Immunology		
TP	6.9	g/dL	CRP	0.02	mg/dL
Alb	4.3	g/dL			
AST	15	U/L	Hematology		
ALT	17	U/L	WBC	9920	/uL
Total Bil	0.69	mg/dL	Hb	14.8	g/dL
LDH	187	U/L	Plt	259	10^3^/uL
ALP	66	U/L			
Crea	0.77	mg/dL	Tumor Markers		
UN	13.6	mg/dL	CEA	4.46	ng/mL
Na	141	mmol/L	CA19-9	6.61	U/mL
K	4.3	mmol/L			
Cl	104	mmol/L			

CRP: C-reactive protein; CEA: carcinoembryonic antigen; CA19-9: cancer antigen 19-9.

**Table 2:** Changes in serum albumin levels. The cycle number indicates the number of administration cycles of the zolbetuximab regimen.

At Time	Alb (g/dL)
Baseline	4.3
S11 + Oxaliplatin	4.4
Cycle 1	4.2
Cycle 2	3.9
Cycle 3	3.9
Cycle 4	4.0
Cycle 5	3.5
Cycle 6	3.6
Surgery	3.3
After Surgery	3.3
Cycle 7	3.8
Cycle 8	3.7
Cycle 9	3.5

## Data Availability

The original data presented in this study are available on reasonable request from the corresponding author. The data are not publicly available due to privacy concerns.

## Abbreviations

The following abbreviations are used in this manuscript:

CLDN18.2	Claudin 18.2
HER2	Human Epidermal Growth Factor Receptor Type 2
PD-L1	Program Death Ligand-1
IHC	Immunohistochemistry
CTCAE	Common Terminology Criteria for Adverse Events

## References

[1] Japanese Gastric Cancer Association (2025). Gastric Cancer Treatment Guidelines.

[2] National Cancer Center Japan, Cancer Registry and Statistics, Cancer Information Services. Hospital-Based Cancer Registry of Designated Cancer Care Hospitals, 2014–2015 5-Year Survival Rate Report. https://ganjoho.jp/public/qa_links/report/hosp_c/hosp_c_reg_surv/index.html.

[3] Bang Y.J., Van Cutsem E., Feyereislova A., Chung H.C., Shen L., Sawaki A., Lordick F., Ohtsu A., Omuro Y., Satoh T. (2010). Trastuzumab in combination with chemotherapy versus chemotherapy alone for treatment of HER2-positive advanced gastric or gastro-oesophageal junction cancer (ToGA): A phase 3, open-label, randomised controlled trial. Lancet.

[4] Janjigian Y.Y., Shitara K., Moehler M., Garrido M., Salman P., Shen L., Wyrwicz L., Yamaguchi K., Skoczylas T., Campos Bragagnoli A. (2021). First-line nivolumab plus chemotherapy versus chemotherapy alone for advanced gastric, gastro-oesophageal junction, and oesophageal adenocarcinoma (CheckMate 649): A randomised, open-label, phase 3 trial. Lancet.

[5] Rha S.Y., Oh D.Y., Yañez P., Bai Y., Ryu M.H., Lee J., Rivera F., Alves G.V., Garrido M., Shiu K.K. (2023). Pembrolizumab plus chemotherapy versus placebo plus chemotherapy for HER2-negative advanced gastric cancer (KEYNOTE-859): A multicentre, randomised, double-blind, phase 3 trial. Lancet Oncol..

[6] Pellino A., Brignola S., Riello E., Niero M., Murgioni S., Guido M., Nappo F., Businello G., Sbaraglia M., Bergamo F. (2021). Association of CLDN18 Protein Expression with Clinicopathological Features and Prognosis in Advanced Gastric and Gastroesophageal Junction Adenocarcinomas. J. Pers. Med..

[7] Niimi T., Nagashima K., Ward J.M., Minoo P., Zimonjic D.B., Popescu N.C., Kimura S. (2001). Claudin-18, a Novel Downstream Target Gene for the T/EBP/NKX2.1 Homeodomain Transcription Factor, Encodes Lung- and Stomach-Specific Isoforms through Alternative Splicing. Mol. Cell. Biol..

[8] Kubota Y., Kawazoe A., Mishima S., Nakamura Y., Kotani D., Kuboki Y., Bando H., Kojima T., Doi T., Yoshino T. (2023). Comprehensive Clinical and Molecular Characterization of Claudin 18.2 Expression in Advanced Gastric or Gastroesophageal Junction Cancer. ESMO Open.

[9] Qi C., Liu C., Peng Z., Zhang Y., Wei J., Qiu W., Zhang X., Pan H., Niu Z., Qiu M. (2025). Claudin-18 Isoform 2-Specific CAR T-Cell Therapy (SATRI-CEL) versus Treatment of Physician’s Choice for Previously Treated Advanced Gastric or Gastro-oesophageal Junction Cancer (CT041-ST-01): A Randomised. Lancet.

[10] Shitara K., Xu R.H., Ajani J.A., Moran D., Guerrero A., Li R., Pavese J., Matsangou M., Bhattacharya P., Ueno Y. (2024). Global Prevalence of Claudin 18 Isoform 2 in Tumors of Patients with Locally Advanced Unresectable or Metastatic Gastric or Gastroesophageal Junction Adenocarcinoma. Gastric Cancer.

[11] Sahin U., Koslowski M., Dhaene K., Usener D., Brandenburg G., Seitz G., Huber C., Türeci O. (2008). Claudin-18 Splice Variant 2 Is a Pan-Cancer Target Suitable for Therapeutic Antibody Development. Clin. Cancer Res..

[12] Shitara K., Lordick F., Bang Y.J., Enzinger P., Ilson D., Shah M.A., Van Cutsem E., Xu R.H., Aprile G., Xu J. (2023). Zolbetuximab plus mFOLFOX6 in Patients with CLDN18.2-Positive, HER2-Negative, Untreated, Locally Advanced Unresectable or Metastatic Gastric or Gastro-oesophageal Junction Adenocarcinoma (SPOTLIGHT): A Multicentre, Randomised, Double-Blind, Phase 3 Trial. Lancet.

[13] Shah M.A., Shitara K., Ajani J.A., Bang Y.J., Enzinger P., Ilson D., Lordick F., Van Cutsem E., Gallego Plazas J., Huang J. (2023). Zolbetuximab plus CAPOX in CLDN18.2-Positive Gastric or Gastroesophageal Junction Adenocarcinoma: The Randomized, Phase 3 GLOW Trial. Nat. Med..

[14] Kwak Y., Kim T.Y., Nam S.K., Hwang H.J., Han D., Oh H.J., Kong S.H., Park D.J., Oh D.Y., Lee H.J. (2025). Clinicopathologic and Molecular Characterization of Stages II–IV Gastric Cancer with Claudin 18.2 Expression. Oncologist.

[15] Yakuwa E., Shoji Y., Oizumi T., Kobayashi Y., Motoishi T., Katagiri T., Suzuki S. (2025). Safety and Feasibility of Outpatient Zolbetuximab Administration in Community Cancer Care: A Mixed-Methods Analysis. In Vivo.

[16] Yanagimoto Y., Yamamoto K., Hara K., Masuike Y., Ushimaru Y., Kitamura M., Honma K., Matsuura N., Sugase T., Kanemura T. (2025). Two Cases of Protein-Losing Enteropathy Induced by Zolbetuximab in Patients with Unresectable Advanced Gastric Cancer. Jpn. J. Clin. Oncol..

[17] Shitara K., Shah M.A., Lordick F., Van Cutsem E., Ilson D.H., Klempner S.J., Kang Y.K., Lonardi S., Hung Y.P., Yamaguchi K. (2024). Zolbetuximab in Gastric or Gastroesophageal Junction Adenocarcinoma. N. Engl. J. Med..

[18] Goyal A., Langer J.C., Zutter M., Swanson P., Kraus M.D., Bartlett N., Shackelford G.D., Longtine J.A., Perlmutter D.H. (1999). Primary gastric plasmacytoma: A rare cause of hypertrophic gastritis in an adolescent. J. Pediatr. Gastroenterol. Nutr..

[19] Seo J.Y., Kim D.H., Ahn J.Y., Choi K.D., Kim H.J., Na H.K., Lee J.H., Jung K.W., Song H.J., Lee G.H. (2024). Differential Diagnosis of Thickened Gastric Wall between Hypertrophic Gastritis and Borrmann Type 4 Advanced Gastric Cancer. Gut Liver.

